# Expression Characterization of Flavonoid Biosynthetic Pathway Genes and Transcription Factors in Peanut Under Water Deficit Conditions

**DOI:** 10.3389/fpls.2021.680368

**Published:** 2021-06-18

**Authors:** Ghulam Kubra, Maryam Khan, Faiza Munir, Alvina Gul, Tariq Shah, Adil Hussain, David Caparrós-Ruiz, Rabia Amir

**Affiliations:** ^1^Atta-Ur-Rahman School of Applied Biosciences (ASAB), National University of Sciences and Technology (NUST), Islamabad, Pakistan; ^2^Department of Agronomy, Faculty of Crop Production Sciences, University of Agriculture Peshawar, Peshawar, Pakistan; ^3^College of Agriculture, Michigan State University, East Lansing, MI, United States; ^4^Department of Agriculture, Abdul Wali Khan University, Mardan, Pakistan; ^5^Centre for Research in Agricultural Genomics (CRAG), Consortium CSIC-IRTA-UAB-UB, Barcelona, Spain

**Keywords:** drought, flavonoid, peanut (*Arachis hypogaea* L), expression profiling, transcription factor

## Abstract

Drought is one of the hostile environmental stresses that limit the yield production of crop plants by modulating their growth and development. Peanut (*Arachis hypogaea*) has a wide range of adaptations to arid and semi-arid climates, but its yield is prone to loss due to drought. Other than beneficial fatty acids and micronutrients, peanut harbors various bioactive compounds including flavonoids that hold a prominent position as antioxidants in plants and protect them from oxidative stress. In this study, understanding of the biosynthesis of flavonoids in peanut under water deficit conditions was developed through expression analysis and correlational analysis and determining the accumulation pattern of phenols, flavonols, and anthocyanins. Six peanut varieties (BARD479, BARI2011, BARI2000, GOLDEN, PG1102, and PG1265) having variable responses against drought stress have been selected. Higher water retention and flavonoid accumulation have been observed in BARI2011 but downregulation has been observed in the expression of genes and transcription factors (TFs) which indicated the maintenance of normal homeostasis. ANOVA revealed that the expression of flavonoid genes and TFs is highly dependent upon the genotype of peanut in a spatiotemporal manner. Correlation analysis between expression of flavonoid biosynthetic genes and TFs indicated the role of *AhMYB111* and *AhMYB7* as an inhibitor for *AhF3H* and *AhFLS*, respectively, and *AhMYB7, AhTTG1*, and *AhCSU2* as a positive regulator for the expression of *Ah4CL, AhCHS, and AhF3H*, respectively. However, *AhbHLH* and *AhGL3* revealed nil-to-little relation with the expression of flavonoid biosynthetic pathway genes. Correlational analysis between the expression of TFs related to the biosynthesis of flavonoids and the accumulation of phenolics, flavonols, and anthocyanins indicated coregulation of flavonoid synthesis by TFs under water deficit conditions in peanut. This study would provide insight into the role of flavonoid biosynthetic pathway in drought response in peanut and would aid to develop drought-tolerant varieties of peanut.

## Introduction

Peanut (*Arachis hypogaea*) is a dryland oilseed crop cultivated worldwide for its high nutritional and commercial value (Wang et al., 2008; Hou et al., 2017). It is cultivated in arid and semi-arid areas that are susceptible to drought stress, which exerts adverse effects on photosynthesis, mineral nutrition, plant metabolism, development, and ultimately crop production (Reddy et al., 2003; Kambiranda et al., 2011). Different plants use different approaches to overcome water shortage for their survival, for instance, drought avoidance and drought tolerance. Plant species opt for a strategy depending on two parameters, intensity and exposure time to drought, and it also includes the ability of that plant to accomplish molecular, biochemical, and physiological variations (Xoconostle-Cazares et al., 2010).

In Arabidopsis, a high level of flavonols in roots of drought-treated Arabidopsis seedlings is associated with the upregulation of the *Flavonol Synthase 1 (AtFLS1)* gene in roots (Nguyen et al., 2016). In tea plants, a significant number of differentially expressed genes are predominantly enriched in volatile compounds, including flavonoids, *Flavonoid 3*′*,5*′*-Hydroxylase* (F3′5′H), *Flavonol Synthase* (FLS), *Flavanone 3-Hydroxylase* (F3H), in response to drought stress (Zheng et al., 2016). It was found that the level of flavonoids in *Triticum aestivum* leaves increases during drought treatment (Ma et al., 2014). Drought conditions can also promote the expression of some flavonoid biosynthetic genes in *Scutellaria baicalensis Georigi* roots (Yuan et al., 2012).

Flavonoids are low molecular weight polyphenolic metabolites that are widely distributed and have distinct biological activities in plants. Flavonoids contribute to plant responses to severe abiotic stresses and have a major role in cell differentiation, growth, and defense signaling (Ma et al., 2014; Khalid et al., 2019). Based on the sites and number of hydroxyl groups, flavonoids are grouped into flavonols, flavones, isoflavones, flavanones, flavonols, and anthocyanidins (Iwashina, 2000; Khalid et al., 2019). In plants, flavonoids are synthesized through the phenylpropanoid pathway. The first step of the pathway is comprised of three enzymes, *Phenylalanine Ammonia Lyase, 4-Coumarate-3-Hydroxylase* (C4H), and *4-Coumaroyl: CoA Ligase* (4CL) that is shared with the production of other phenylpropanoid compounds, such as the lignin polymer. Then, the specific flavonoid pathway involves the following enzymes: comprises the involvement of *Chalcone Synthase* (CHS), *Chalcone Isomerase* (CHI), *Flavone Synthase* (FS), and *Dihydroflavonol-4-Reductase* (DFRA) (Winkel-Shirley, 2001).

Peanuts, other than beneficial fatty acids and micronutrients, harbor various bioactive compounds, including saponins, terpenoids, quinones, resveratrol, flavonoids, alkaloids, polyphenolics, phlobatannins, tocopherols, tannins, cardiac glycosides, and phenolic compounds that impart beneficial physicochemical and nutritional properties to it (Sim et al., 2012; Rebaya et al., 2014; Prabasheela et al., 2015). These secondary metabolites of plants are the target of many pharmaceutical and nutraceuticals due to their health-promoting properties (Chang et al., 2002). Among them, flavonoids hold a prominent position as they are potential antioxidants in plants and protect them from oxidative stress. Globally, breeders are trying to develop peanut varieties as functional food with enhanced flavonoid contents. Peanuts were reported with the presence of various forms of flavonoids including C-glycoside flavone, flavonol, dihydroflavonol, flavonone and 5,7-dimethoxyisoflavone, and dihydroquercetin. The presence of these flavonoids can improve the tolerance of peanuts to biotic and abiotic stresses (Mabry et al., 1970; Daigle et al., 1983). The genome of peanut has been recently sequenced, but its characterization is yet to be explored. The flavonoid biosynthetic pathway in peanut needs further research and opens many horizons to develop an understanding of this pathway.

This study has been designed to unravel the flavonoid biosynthetic pathway in peanuts under water deficit conditions. Flavonoid biosynthetic pathway genes and TFs associated with flavonoid production have been selected for expression characterization under water deficit conditions in peanuts. Six peanut varieties (BARD479, BARI2011, BARI2000, GOLDEN, PG1102, PG1265) were selected based on their yield potential and their performance against drought stress to determine the role of flavonoids in drought stress tolerance. Moreover, correlational analysis was performed to determine the interdependency of the expression of flavonoid biosynthetic pathway genes and TFs with an accumulation of phenolics, flavonols, and anthocyanins under water deficit conditions in peanut. The study would provide insight into the role of flavonoid biosynthetic pathway in drought response in peanut and would aid to develop drought-tolerant varieties of peanut. Moreover, the study provides reference data for the flavonoid biosynthetic pathway engineering for higher production of flavonoids for industrial use.

## Materials and Methods

### Plant Material and Treatments

Six varieties of peanuts (BARD479, BARI2011, BARI2000, GOLDEN, PG1102, and PG1265) were obtained from the Oilseed department of the National Agricultural Research Centre (NARC), Pakistan after obtaining permission from an authorized person. Varieties were selected based on their yield potential and their performance against drought stress. BARD479, BARI2011, BARI2000, and GOLDEN are popular cultivars of peanut (yield potential of BARD479 = 4,000 Kg/ha, yield potential of BARI2011 = 6,300 Kg/ha, yield potential of BARI2000 = 4,000 Kg/ha, and yield potential of Golden = 4,100 Kg/ha) (Saeed and Hassan, 2009; Naeem-ud-Din et al., 2012). Among them BARI2011, BARI2000, and GOLDEN are drought tolerant while the remaining three varieties (PG1102, PG1265, and BARD479) are drought-sensitive. All the experiments were performed according to the guidelines provided by the Coordinated Framework for Regulation of Biotechnology and institutional review board (IRB), ASAB, NUST, Pakistan. Healthy seeds were de-husked and surface sterilized by soaking in 70% ethanol for 1 min and 3.5% bleach (NaOCl) solution for 5 min followed by washing with sterile distilled water several times (Iqbal et al., 2011). Seeds of all the varieties were chilled at 4°C for 2 days to break dormancy and ensure uniform germination. Six varieties of peanuts were sown in pots (10.16×6.35 cm) filled with peat moss (three seeds per pot; five pots for control and five for drought stress for each peanut variety). Optimum growth conditions of 16/8 h of light and dark conditions at 25–30°C were maintained according to the recommendations of Pruthvi et al. (2014). The field capacity of potting media was measured by determining the difference between the saturated wet and completely dried weight of potting media as explained by Lopez and Barclay (2017). After germination, plants were watered regularly for 2 weeks according to field capacity. Plants at the same developmental stage and similar height were selected for treatment. Each variety was divided into control group (*N* = 9) and treated group (*N* = 9). The control group was watered regularly, whereas, water deficit conditions were given to the treated group for 2 weeks. Symptoms of water deficit conditions were observed regularly, and samples were collected on day 14 in plastic tubes followed by quick freezing in liquid nitrogen. Samples were stored at −80°C until further analysis.

### Relative Water Content (RWC)

A fully developed third leaf from the top was used for RWC measurement. The fresh weight (FW) of the leaves from each variety was recorded just after sampling the leaves. Turgid weight (TW) was obtained after soaking leaves in distilled water in beakers for 24 h at room temperature and under low light conditions. After soaking, the leaves were quickly blot dried with tissue paper for determining TW. Dry weight (DW) of the leaves was obtained after oven drying the leaf samples for 72 h at 60°C. RWC was calculated in three replicates by following the method described by Sumithra et al. (2006). RWC (%) = [(FW-DW)/(TW-DW)] × 100.

### Determination of Anthocyanin Content

Total anthocyanin content was determined according to Laitinen et al. (2008). Leaf tissues (100 mg) were extracted using 1 ml of extraction solvent (methanol, water, hydrochloric acid, 7:2:1) at 4°C for 20 h and centrifuged for 20 min at 10,000 rpm at 4°C. The absorbance of the supernatants was measured at 530 nm. The total anthocyanin content was expressed as cyaniding-3-glucoside equivalents from the calibration plot (y = 0.0658x – 0.0035, R^2^ = 0.9979). All determinations were carried out in triplicate.

### Determination of Total Flavonol Content

Total flavonols were determined according to the method explained by Chang et al. (2002). Leaf tissues (100 mg) were extracted in 80% methanol at 4°C for 2 h. After centrifugation, aliquots of supernatant were taken and mixed with 2 ml of methanol and sequentially mixed with 0.1 ml of aluminum chloride (10% water solution), 0.1 ml of 1 M K-acetate, and 2.8 ml of distilled water. After 30 min incubation at room temperature, absorbance was recorded at 415 nm. The concentration of total flavonol content in the test sample was calculated from the calibration plot (y = 0.0081x, R^2^ = 0.9559) and expressed as μg quercetin equivalent (QE)/g of dried plant material. All determinations were carried out in triplicate.

### Determination of Total Phenolic Content

Total phenolics were determined using a modified Folin Ciocalteu colorimetric method as described by Singleton and Rossi (1965). Fresh leaf tissues (100 mg) were extracted using 1 ml of ethanol (80%), incubated for 2 h at 4°C in dark, and then centrifuged to remove cell debris. Aliquots of supernatant were made up to a volume of 3 ml with distilled water. Then, 0.5 ml of Folin Ciocaltaeu reagent (1:1 with water) and 2 ml of Na_2_CO_3_ (20%) were added. The solution was incubated at 45°C for 15 min, cooled to room temperature, and the absorbance was measured at 650 nm. The phenolic content was calculated as a gallic acid equivalents GAE/g of dry plant material based on the standard curve of gallic acid (y = 0.0181x + 0.0033, R^2^ = 0.995). All determinations were carried out in triplicate.

### RNA Extraction and cDNA Synthesis

Total RNA was extracted from leaves and roots samples by using the Promega Maxwell^®^ 16 LEA Plant RNA kit (Promega, Fitchburg, USA). The quality and concentration of RNA were determined by NanoDrop^™^ 2000/2000c Spectrophotometers (Thermo Scientific, Wilmington, DE, USA). Extracted RNA was reverse transcribed with SuperscriptTM III Reverse Transcriptase with oligo (dT) 18 as the primer to synthesize the first-strand cDNA.

### Gene Expression Analysis

The flavonoid biosynthetic pathway in Arabidopsis has been used as a reference to identify genes and TFs associated with flavonoid synthesis in peanuts. A total of nine genes encoding biosynthetic enzymes (*AhC4H, Ah4CL, AhCHS, AhCHI, AhF3H, AhFLS, AhLAR, AhDFR, and AhANS*) and eight TFs (*AhMYB111, AhMYB123, AhMYB7, AhTTG1, AhbHLH, AhGL3, AhCOP1*, and *AhCSU2*) has been selected from peanut for expression analysis using a Fluidigm 96.96 Dynamic array IFC (Integrated Fluidic Circuits) (IntegraGen, Evry France). Dynamic arrays were performed in FLUIDIGM-BioMark System (IntegraGen, Evry France) (quantitative reverse transcription-PCR, qRT-PCR) for plant samples. C_t_ values were calculated by Fluidigm Real-time PCR analysis software. Linear derivatives and automatic detector methods were set for baseline correction. The expression efficiencies were calculated with LinRegPCR (12.x) software, and ΔC_t_ was calculated according to the protocol described by Karlen et al. (2007). Primers for genes [*Ah4CL3, AhC4H, AhCHS, AhCHI, AhF3H, AhFLS, AhLAR* ***(****Leucoanthocyanin reductase****)****, AhDFR*, and *AhANS* ***(****Anthocyanidin synthase****)***] and transcriptional regulators (*AhMYB111, AhMYB123, AhMYB7, AhTTG1, AhbHLH, AhGL3, AhCOP1*, and *AhCSU2*) were designed using the bioinformatics tool, https://www.ncbi.nlm.nih.gov/tools/primer-blast/, aiming at an amplicon length of 90–200 bp. Actin was used as the reference gene for reverse transcription-quantitative PCR (RT-qPCR) data normalization. Primer sequences are given in the Supplementary Material.

### Statistical Analysis

The statistical analysis of univariate data, including gene and TFs expression profiles, was performed in Rstudio software. The normality of the data was assessed using the variance homoscedasticity Bartlett test and Shapiro test of normality using default functions of R. Normally distributed data were analyzed with the construction of an ANOVA model, significance was assessed using the D'Agostino test of skewness on the residual variance (Komsta and Novomestky, 2015), followed by a *post-hoc* Tukey's Honest Significant Detection test (Tukey's HSD, *p* < 0.05) (De Mendiburu, 2014). Non-parametric data were analyzed with a Kruskal–Wallis test followed by a *post-hoc* False Discovery Rate test correction to account for multiple testing (FDR, *p* < 0.05, package “*agricolae*”). Correlational analysis among various selected variables was assessed through Pearson's correlation test using GraphPad Prism^®^ version 5.

## Results

### Variation in Metabolites Following Water Deficit Conditions

As flavonols act as free radical scavengers and play an imperative role in countering abiotic stress, the flavonol content of the leaves collected from seedlings of several peanut varieties grown under water deficit conditions was examined. The analysis revealed that the flavonol content in leaves was upregulated in different varieties of peanuts under water deficit conditions (Table 1). The interactive effect between peanut varieties and water deficit conditions was also significant (Table 1).

**Table 1:** ANOVA for metabolites of peanut genotypes under drought stress.

**Factor**	**Leaf Flavonol**	**Leaf Anthocyanin**	**Leaf Phenol**	**Root Flavonol**	**Root Anthocyanin**	**Root Phenol**	**RWC**	**DW**	**FW**
Varieties (V)	343.59^***^	114.07^***^	57.08^***^	6.23^**^	319.20^***^	203.81^***^	39.08^***^	3.18	2.02
Drought stress (D)	24.58^***^	55.71^***^	18.11^***^	7.10^***^	42.94^***^	11.27^***^	0.37	12.09^***^	4.85^***^
Interaction (VxD)	10.27^***^	3.75^**^	3.99^**^	10.33^***^	7.43^***^	10.80^***^	0.71	0.0003^***^	3.55^**^
R^2^	10.6	4.5	10.99	44.65	6.57	17.78	5.15	29.25	26.05

** *P < 0.01*.

*** *P < 0.001*.

*F values are given, with asterisks indicating the significance of effects. RWC, Relative water content; DW, Dry weight; FW, Fresh weight. The last row provides the standard R^2^ values. The total number of degrees of freedom is 41*.

**Figure 1:**
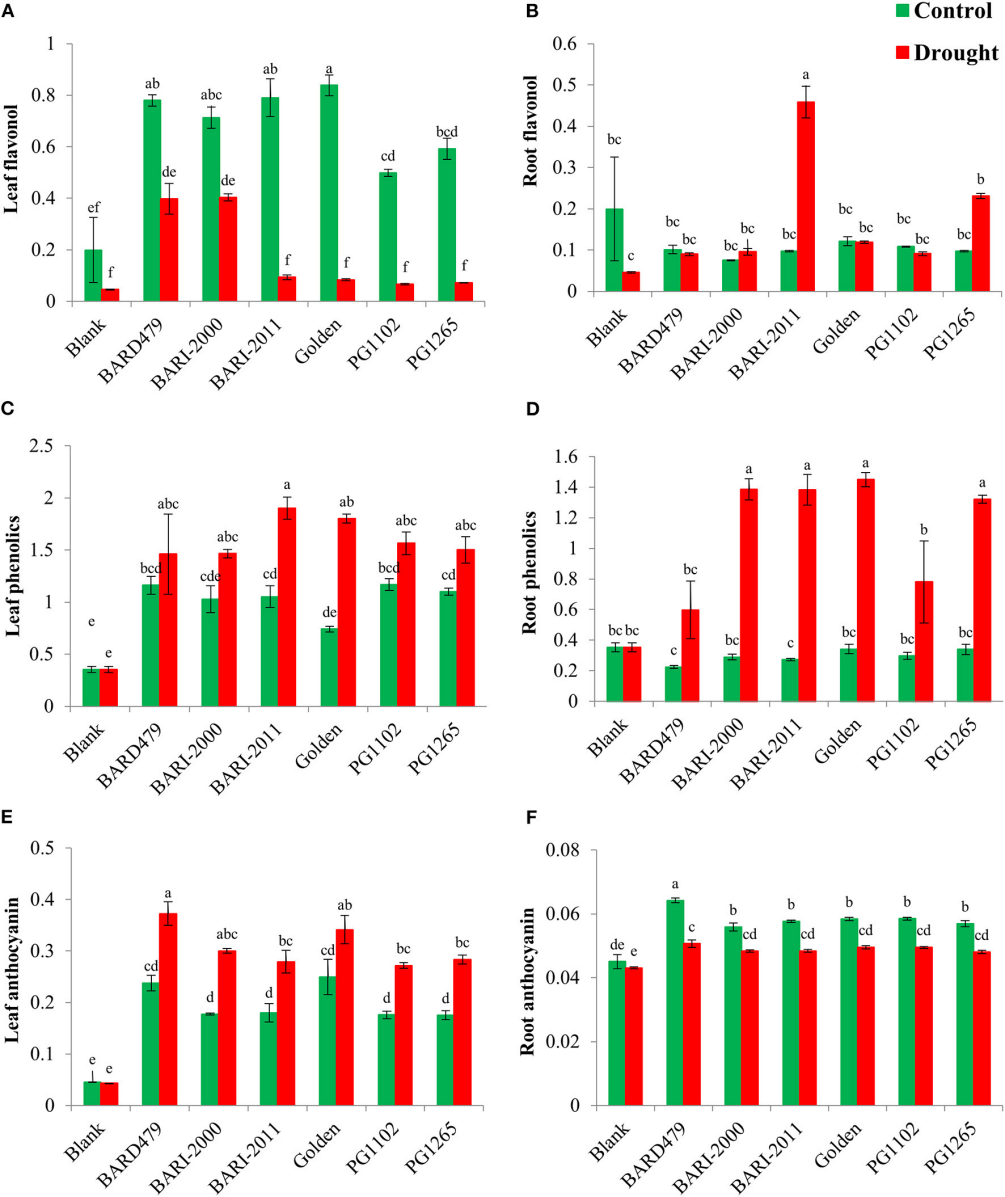
Metabolites content accumulation in peanut genotypes under drought stress. The panel shows the following: the leaf flavonol **(A)**, the root flavonol **(B)**, the leaf phenolics **(C)**, the root phenolics **(D)**, the leaf anthocyanin **(E)**, and the root anthocyanin **(F)**. Data are shown as the interaction between the genotypes of peanuts and the drought applied. Significance was inferred either with an ANOVA under the Tukey's HSD *post-hoc* test for normally distributed data (Honest Significant Detection, *p* < 0.05). Non-parametric data were analyzed with a Kruskal–Wallis test under False Discovery Rate *post-hoc* correction (FDR, *p* < 0.05).

Maximum upregulation of flavonol content was observed in the leaves of GOLDEN, BARI2011, BARD479, and BARI2000 as compared with PG1265 and PG1102 under control conditions (Figure 1A). Under water deficit conditions, BARI2000 resulted in maximum upregulation followed by BARD479 while BARI2011, GOLDEN, PG1265, and PG1102 have minimum flavonol content (Figure 1A). ANOVA showed that flavonol content in roots of all the varieties was significantly affected by water deficit conditions and the interaction was also found to be statistically significant (Table 1).

Concerning peanut roots, BARI2011 and PG1265 varieties increased their flavonol content when plants were subjected to water deficit conditions compared with control plants (Figure 1B). The most conspicuous class of flavonoids is anthocyanin, which is induced in plants to resist several abiotic stresses. Water deficit conditions strongly increased anthocyanin content. This result also revealed that anthocyanin in the leaf of different varieties was upregulated under water deficit conditions while the interaction was also found significant (Table 1). The anthocyanin content was found to be the same for all varieties of peanuts. However, significant upregulation of anthocyanin content was observed in the GOLDEN and the BARD479 varieties under control conditions (Figure 1E). BARD479 variety has more anthocyanin followed by GOLDEN, BARI2000, and BARI2011 as compared with other varieties under water deficit conditions (Figure 1E). Anthocyanin content in the roots of peanut varieties differed significantly under water deficit conditions (Table 1). The interactive effect was also found significant (Table 1). More anthocyanin content was accumulated in the roots of the BARD479 variety as compared with others under control (Figure 1F). Under water deficit conditions, BARD479 accumulated more anthocyanin content in their roots which was statistically similar to other varieties (Figure 1F).

The phenolic contents play an important role in neutralizing the harmful reactive oxygen species (ROS) under drought stress and protect plants from oxidative stress. The leaf phenolic content of varieties was significantly upregulated under water deficit conditions (Table 1). The interaction was also found significant (Table 1). More phenolic content was recorded in PG1102 and BARD479, whereas the GOLDEN variety resulted in lower phenolic content under control (Figure 1C). Regarding water deficit conditions, BARI2011 accumulated more phenolic content followed by the GOLDEN variety as compared with other varieties (Figure 1C). The varietal effect was found significant for root phenolic content under water deficit conditions while their interaction was also found to be significant. Under the control condition, the GOLDEN variety resulted in more root phenolic content, but the effect was statistically similar with other varieties (Figure 1D). Golden variety accumulated more phenolics in the roots followed by BARI2000, BARI2011, and PG1265, whereas BARD479 and PG1102 had a significantly lower phenolic content in their roots under water deficit conditions.

### Determination of FW, DW, and RWC of Peanut Varieties Following Water Deficit Conditions

**Figure 2:**
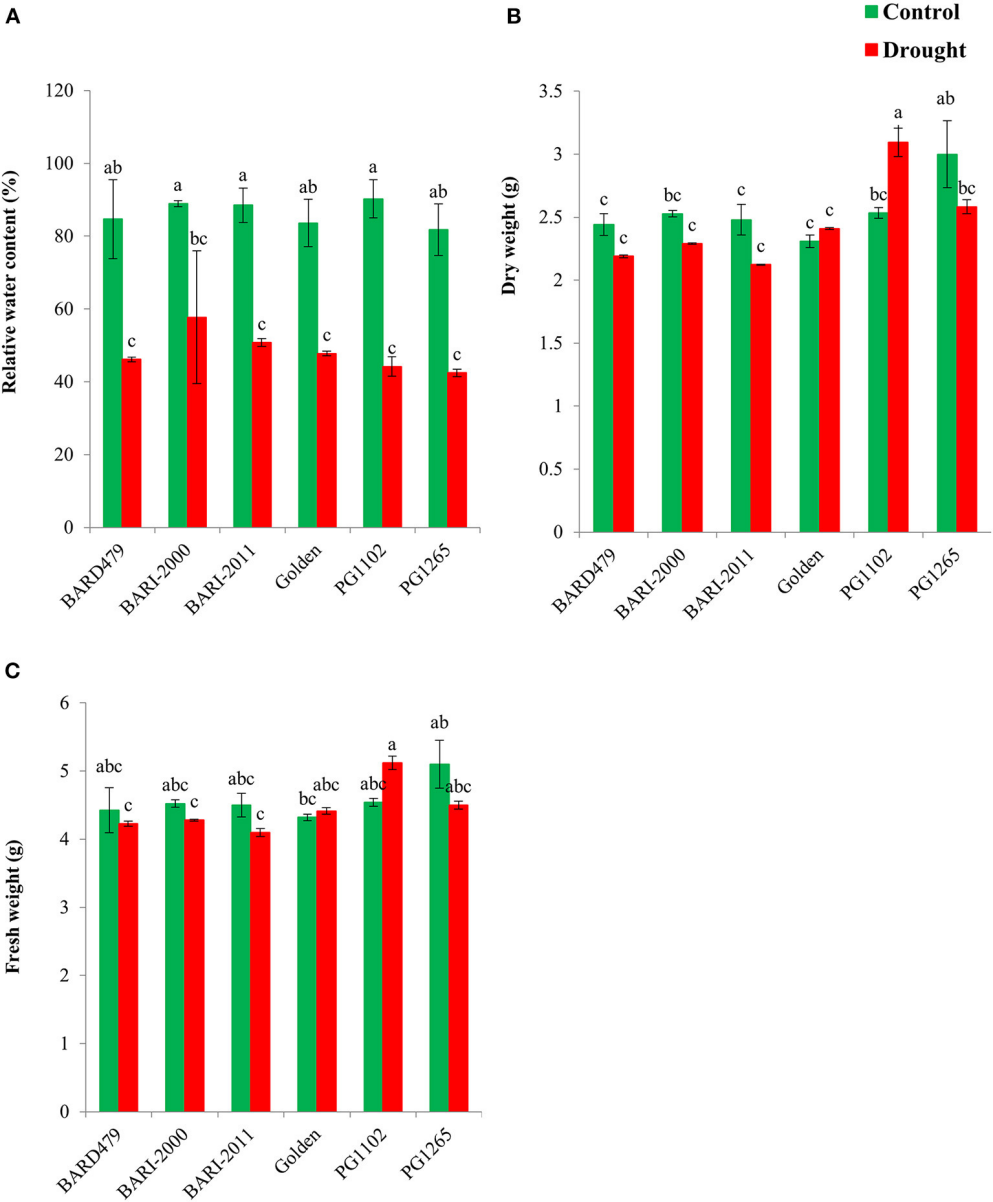
Water status and biomass of genotypes of peanut under drought stress. The panel shows the following: the relative water content (RWC) **(A)**, the dry weight (DW) **(B)**, and the fresh weight (FW) **(C)**. Data are shown as the interaction between the genotypes of peanut and the drought applied. Significance was inferred either with an ANOVA under the Tukey's HSD *post-hoc* test for normally distributed data (Honest Significant Detection, *p* < 0.05). Non-parametric data were analyzed with a Kruskal–Wallis test under False Discovery Rate *post-hoc* correction (FDR, *p* < 0.05).

Relative water content was significantly affected by water deficit conditions, however, the varietal effect and their interaction were found to be non-significant (Table 1). RWC is higher in plants grown in control conditions compared with the ones grown in water deficit conditions (Figure 2A). BARI2011 has maximum RWC under water deficit conditions but the effect was similar to other varieties (Figure 2A). Water deficit conditions had no effect on FW for the majority of peanut varieties (Figure 2C), although, the effect of varieties and their interaction were found significant (Table 1). PG1102 is the only variety that has more FW under water deficit conditions as compared with control plants (Figure 2C). There is no effect of water deficit on DW for the majority of the tested varieties, however, the effect of varieties and their interaction was found significant (Table 1). PG1102 is the only variety that has more DW under water deficit conditions as compared with control plants (Figure 2B).

### Expression Pattern of Genes in Flavonoid Biosynthetic Pathway

**Figure 3:**
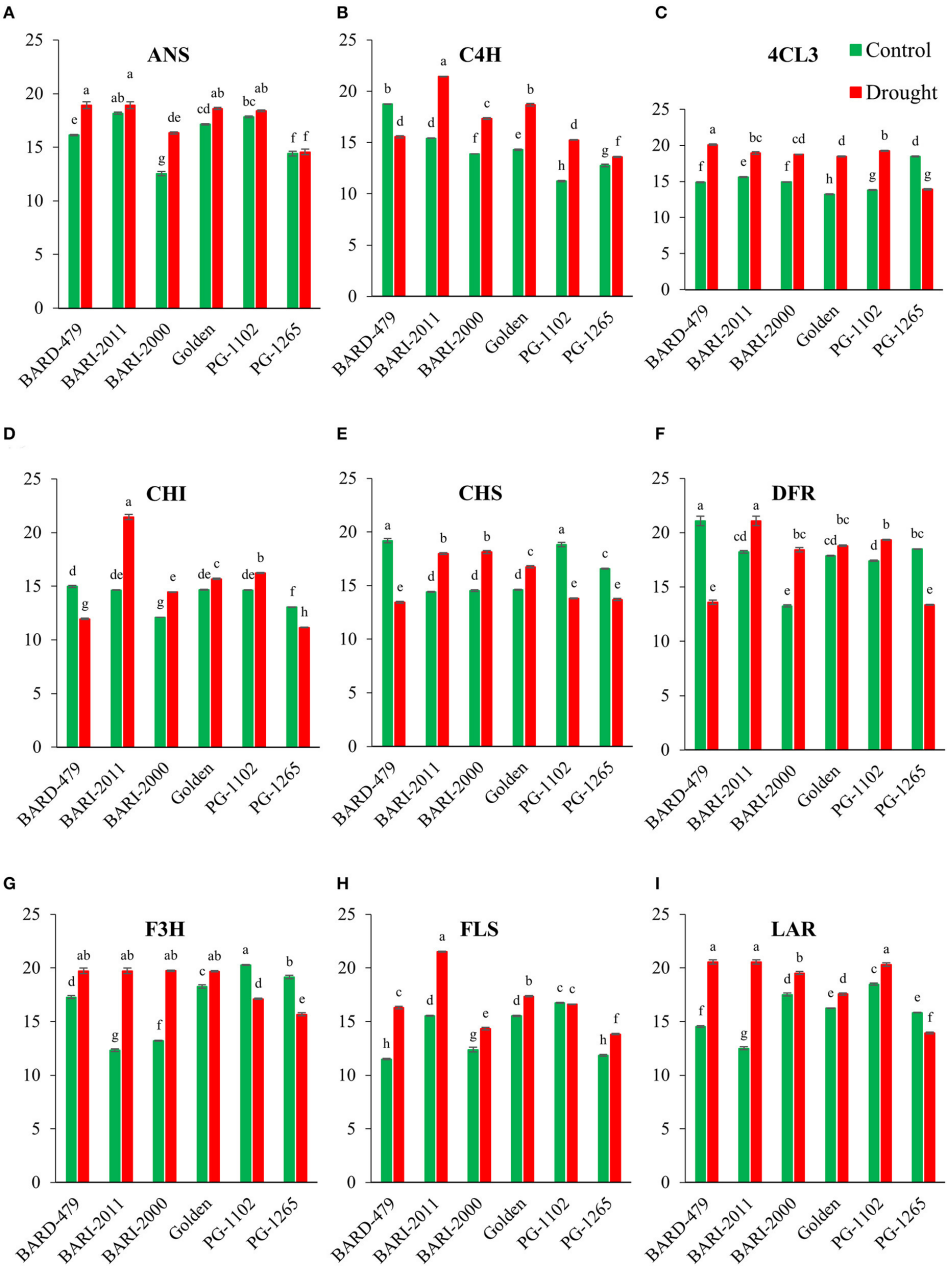
Gene expression profile in leaves of genotypes of peanuts under drought stress. The panel shows the following: the ANS **(A)**, the C4H **(B)**, the 4CL3 **(C)**, the CHI **(D)**, the CHS **(E)**, the DFR **(F)**, the F3H **(G)**, the FLS **(H)**, and the LAR **(I)**. Data are shown as the interaction between the genotypes of peanuts and the drought applied. Significance was inferred either with an ANOVA under the Tukey's HSD *post-hoc* test for normally distributed data (Honest Significant Detection, *p* < 0.05). Non-parametric data were analyzed with a Kruskal–Wallis test under False Discovery Rate *post-hoc* correction (FDR, *p* < 0.05).

The gene expression pattern of early and late genes of flavonoid biosynthetic pathway has been determined in leaves and roots of six different varieties of peanuts under water deficit conditions. A significant inter-varietal difference in the expression of early and late pathway genes has been observed. Under water deficit conditions, the expression of *AhANS* increased significantly in all varieties in comparison to their respective controls with exception of PG1265, which did not show any significant variation in expression in comparison with its control (Figure 3A). *AhC4H* depicted a distinct pattern of expression, under water deficit conditions, the expression was significantly elevated in leaves of all varieties except BARD479 that was significantly downregulated (Figure 3B). The expression of *Ah4CL3* was significantly increased in leaves of all varieties under water deficit conditions except PG1265 which had lower expression in comparison with its respective control (Figure 3C). The expression pattern of *AhCHI* was distinct from *AhCHS*. The untreated plants of BARD479, BARI2011, PG1102, and GOLDEN had similar expression patterns, which did not significantly deviate from water deficit plants of BARI2000. However, the increased expression of *AhCHI* was observed in water deficit plants from BARI2011, BARI2000, PG1102, and GOLDEN varieties whereas the expression of *AhCHI* was significantly downregulated in water deficit plants of BARD479 and PG1265 in comparison with their respective controls (Figure 3D). The expression pattern of *AhCHS* in leaves of untreated plants of BARD479 and PG1102 was significantly elevated in comparison with the plants under water deficit plants and untreated plants of other varieties. However, the expression of *AhCHS* in water deficit, BARD479, PG1102, and PG1265, varieties is reduced compared with their respective controls. The water deficit plants of BARI2011, BARI2000, and GOLDEN have elevated expression, whereas the untreated plants had no significant difference (Figure 3E). The expression of *AhDFR* was significantly elevated in water deficit varieties of BARI2000, BARI2011, PG1102, and GOLDEN in comparison with their respective control, whereas the expression was observed to be significantly decreased in water deficit varieties of BARD479 and PG1265 in comparison with their respective control. However, the untreated plants of BARD479 and PG1265 depicted a significant increase in *AhDFR* expression in comparison with other varieties (Figure 3F). The late pathway genes depicted a distinct expression pattern. Under water deficit conditions, the expression of *AhF3H* was significantly elevated in BARD479, BARI2011, BARI2000, and GOLDEN varieties in comparison with their respective controls, whereas the expression of water deficit varieties of PG1102 and PG1265 had significantly decreased expression. The inter-varietal difference in *AhF3H* expression depicted that expression was not significant in water deficit varieties of BARD479, BARI2011, BARI2000, and GOLDEN but it significantly deviated from PG1102 and PG1265. The untreated plants of BARD479, BARI2011, BARI2000, and GOLDEN had significantly decreased expression but PG1102 and PG1265 had significantly elevated expression in comparison with their respective controls (Figure 3G). The expression of *AhFLS* was significantly increased under water deficit conditions in all varieties with exception of PG1102 since it did not depict a significant difference in comparison with its respective control (Figure 3H). A significant increase in the expression of *AhLAR* was observed in all varieties in comparison with their respective controls except PG1265 that had significantly decreased expression in comparison with its control. The expression of *AhLAR* was significantly increased in untreated BARI2000 and PG1102 in comparison with other varieties (Figure 3I).

**Figure 4:**
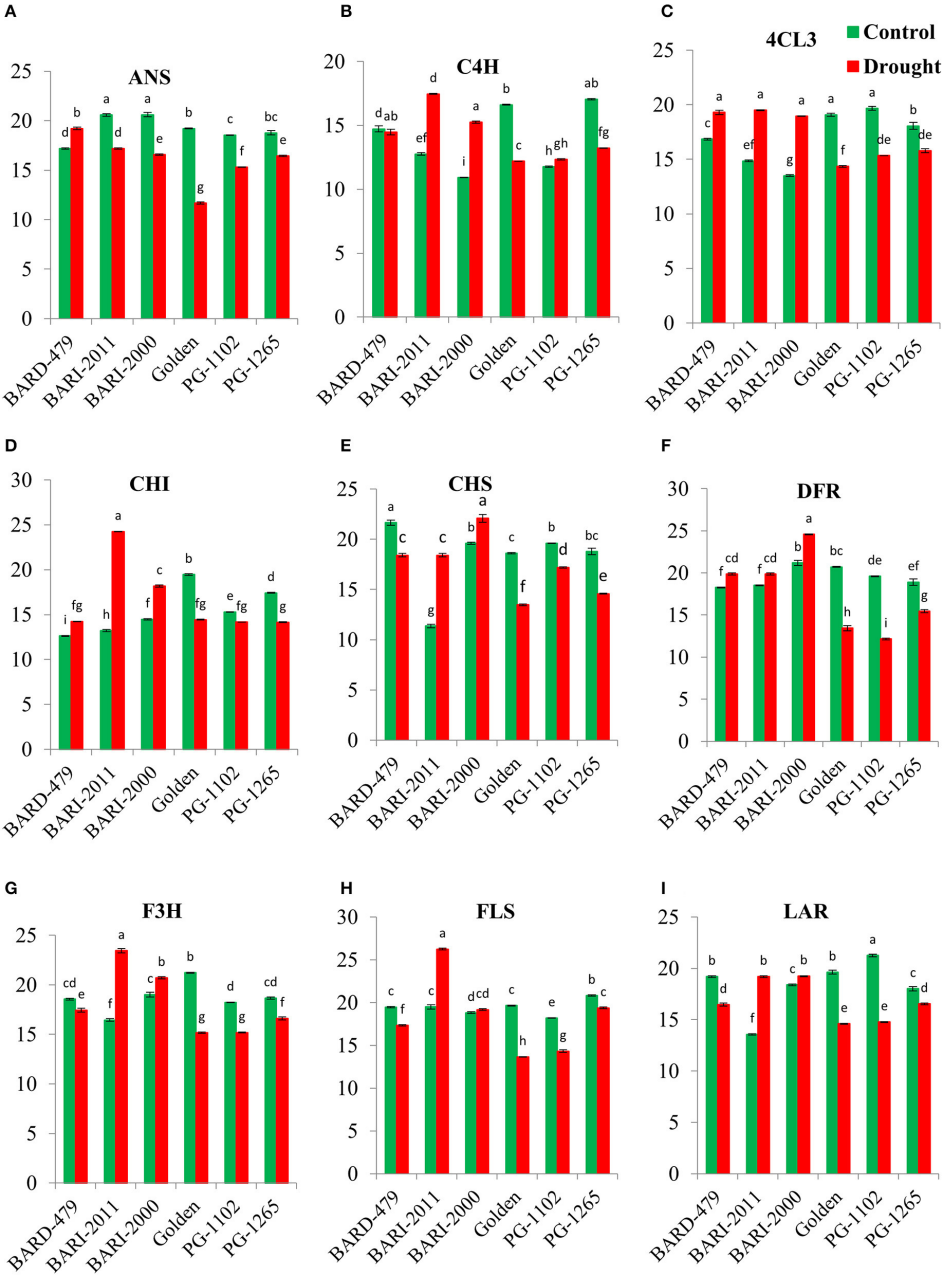
Gene expression profile in roots of genotypes of peanuts under drought stress. The panel shows the following: the ANS **(A)**, the C4H **(B)**, the 4CL3 **(C)**, the CHI **(D)**, the CHS **(E)**, the DFR **(F)**, the F3H **(G)**, the FLS **(H)**, and the LAR **(I)**. Data are shown as the interaction between the genotypes of peanuts and the drought applied. Significance was inferred either with an ANOVA under the Tukey's HSD *post-hoc* test for normally distributed data (Honest Significant Detection, *p* < 0.05). Non-parametric data were analyzed with a Kruskal–Wallis test under False Discovery Rate *post-hoc* correction (FDR, *p* < 0.05).

Under water deficit conditions, the expression of *AhANS* was significantly decreased in all varieties in comparison with their respective controls with exception of BARD479, which had a significant increase in the expression of *AhANS* in comparison with its control (Figure 4A). *AhC4H* depicted a distinct pattern of expression. Under water deficit conditions, its expression was significantly elevated in BARI2000 and BARI2011 varieties, however, the expression was significantly downregulated in Golden, PG1102, and PG1265 in comparison with their respective control. BARD479 did not show significant variation in expression from its control (Figure 4B). The expression of *Ah4CL3* was significantly increased in roots of BARI2000, BARI2011, and BARD479 under water deficit conditions in comparison with their respective control, whereas the expression was observed to be significantly decreased in water deficit Golden, PG1102, and PG1265 varieties in comparison with their respective control (Figure 4C). The significantly increased expression of *AhCHI* was observed in water deficit plants from BARI2011, BARI2000, and BARD479 varieties in comparison with their respective controls but the expression of *AhCHI* was significantly downregulated in water deficit PG1102, GOLDEN, and PG1265 varieties in comparison with their respective controls (Figure 4D). The expression pattern in roots was distinct from the expression in leaves. The expression pattern of *AhCHS* in roots of water deficit BARI2011 and BARI2000 was significantly elevated in comparison to their respective controls. Whereas, the water deficit conditions of BARD479, PG1102, GOLDEN, and PG1265 had significantly decreased expression of *AhCHS* in comparison with their respective controls (Figure 4E). The expression of *AhDFR* was significantly elevated in roots of water deficit BARI2000, BARI2011, and BARD479 in comparison with their respective control whereas the expression was observed to be significantly decreased in water deficit Golden, PG1102, and PG1265 varieties in comparison with their respective control (Figure 4F). The late pathway genes depicted a distinct expression pattern in roots as well. Under water deficit conditions, the expression of *AhF3H* was significantly elevated in BARI2011 and BARI2000 varieties in comparison with their respective controls, whereas the expression of water deficit BARD479, GOLDEN, PG1102, and PG1265 had significantly decreased expression (Figure 4G). The expression of *AhFLS* was significantly decreased under water deficit conditions in all varieties with exception of BARI2011, since it had significantly increased expression in comparison with its respective control. However, BARI2000 did not show any significant difference in comparison with its respective control (Figure 4H). A significant increase in the expression of *AhLAR* was observed in BARI2000 and BARI2011 under water deficit conditions in comparison with their respective controls, whereas all other varieties had significant downregulation in *AhLAR* expression. The expression of *AhLAR* was significantly increased in untreated BARD479, GOLDEN, and PG1102 varieties in comparison with other varieties (Figure 4I).

### Transcription Factors (TFs) Expression Profile in Roots and Leaves of Peanut Genotypes Under Water Deficit Conditions

**Figure 5:**
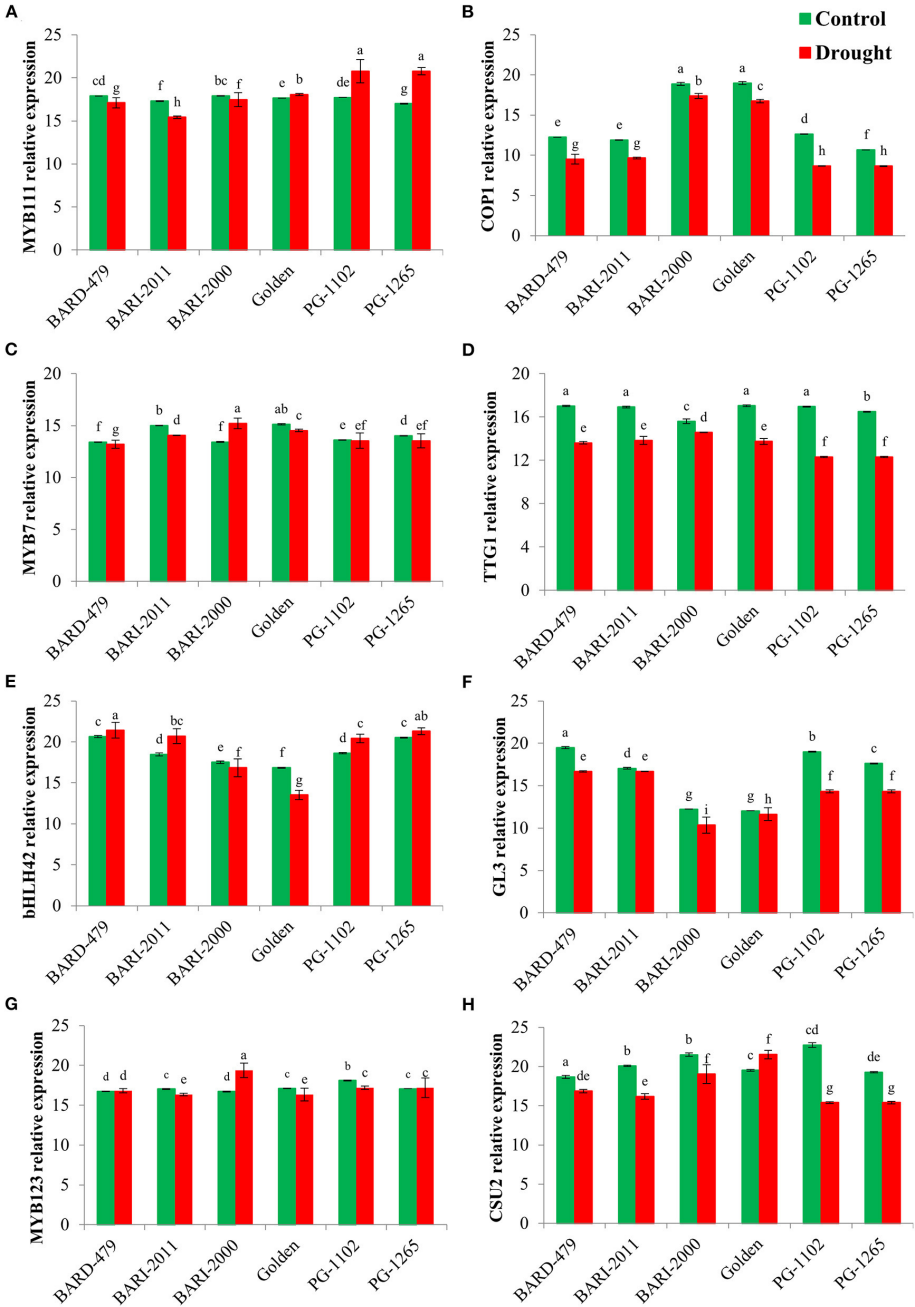
Transcription factors (TFs) expression profile in leaves of genotypes of peanuts under drought stress. The panel shows the following: the MYB111 **(A)**, the COP1 **(B)**, the MYB7 **(C)**, the TTG1 **(D)**, the bHLH42 **(E)**, the GL3 **(F)**, the MYB123 **(G)**, and the CSU2 **(H)**. Data are shown as the interaction between the genotypes of peanut and the drought applied. Significance was inferred either with an ANOVA under the Tukey's HSD *post-hoc* test for normally distributed data (Honest Significant Detection, *p* < 0.05). Non-parametric data were analyzed with a Kruskal–Wallis test under False Discovery Rate *post-hoc* correction (FDR, *p* < 0.05).

**Figure 6:**
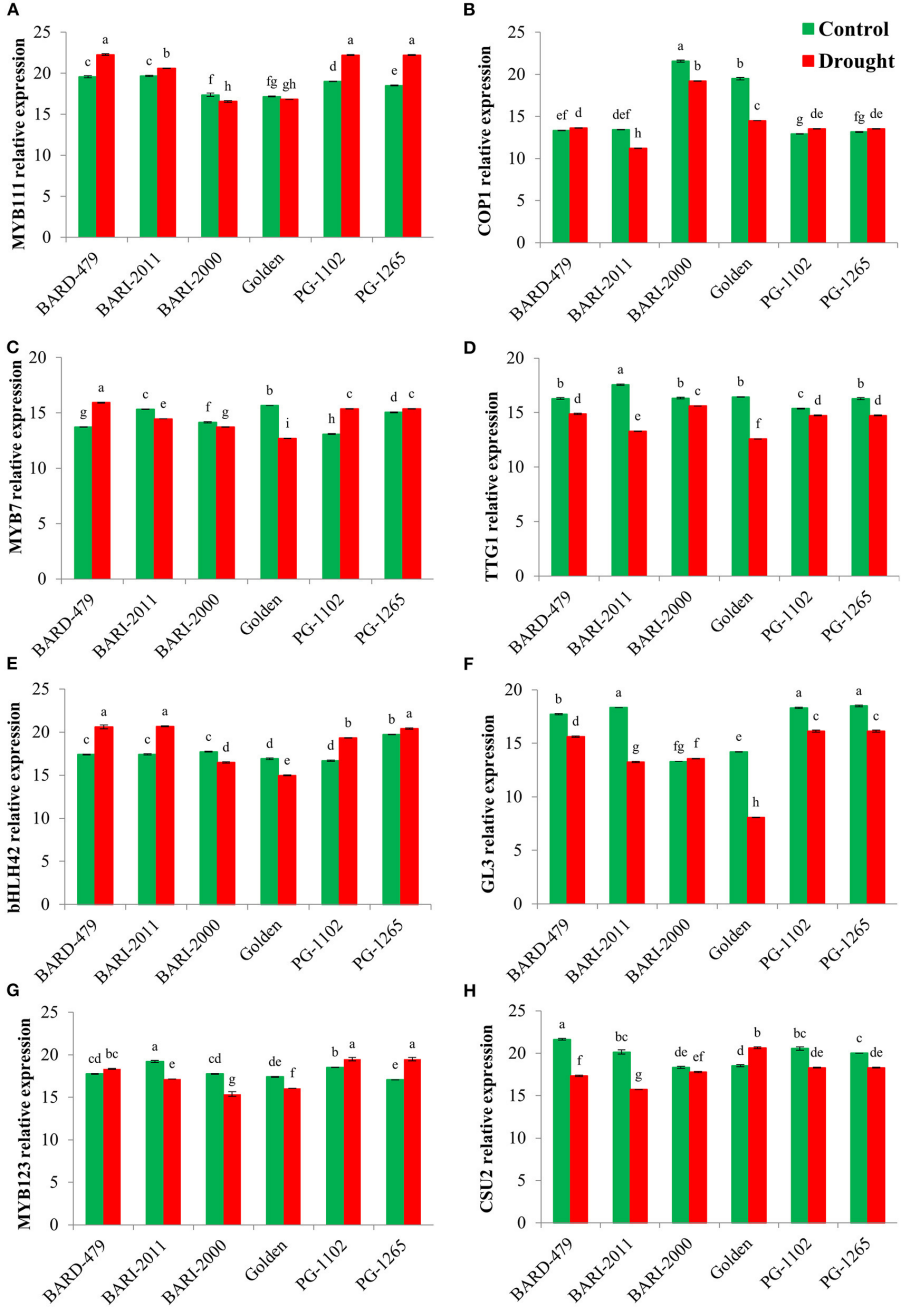
Transcription factors expression profile in roots of genotypes of peanus under drought stress. The panel shows the following: the MYB111 **(A)**, the COP1 **(B)**, the MYB7 **(C)**, the TTG1 **(D)**, the bHLH42 **(E)**, the GL3 **(F)**, the MYB123 **(G)**, and the CSU2 **(H)**. Data are shown as the interaction between the peanut genotypes and the drought applied. Significance was inferred either with an ANOVA under the Tukey's HSD *post-hoc* test for normally distributed data (Honest Significant Detection, *p* < 0.05). Non-parametric data were analyzed with a Kruskal–Wallis test under False Discovery Rate *post-hoc* correction (FDR, *p* < 0.05).

Following the determination of water deficit-mediated variations in the expression of various genes directly involved in secondary metabolite biosynthesis, we checked the expression profiles of various TFs involved in secondary metabolism following the water deficit conditions. *MYB11*, which is a member of the R2R3 factor gene family positively regulates the production of flavonols in plants. The relative expression of *AhMYB111* in the leaves was significantly upregulated in the varieties PG1102, PG1265, and GOLDEN with the maximum expression being recorded in PG1102 and PG1265 followed by the GOLDEN variety (Figure 5A). Furthermore, the highest relative expression of *AhMYB111* in the roots was also recorded in PG1102 and PG1265 along with BARD479, followed by BARI2011 following water deficit conditions. *AhMYB111* expression was significantly downregulated in the roots of BARI2000 and GOLDEN varieties following water deficit conditions (Figure 6A). *AhCOP1* expression was found to be downregulated by water deficit conditions in all the varieties. However, the highest relative expression of *AhCOP1* was recorded in BARI2000 and GOLDEN varieties both under optimal and water deficit conditions, followed by BARD479, and BARI2011 varieties (Figure 5B). In roots, maximum relative *AhCOP1* expression under optimal as well as water deficit conditions was recorded in BARI2000 variety followed by GOLDEN, BARD479, and PG1265 varieties (Figure 6B). In Arabidopsis, *AhMYB7* encodes a repressor of flavonol biosynthesis (Stracke et al., 2010), and its maximum relative expression was recorded in BARI2000 and GOLDEN varieties following the water deficit conditions (Figure 5C). In the roots, however, the maximum relative expression of *AhMYB7* was recorded for BARI2011 and GOLDEN varieties under optimal conditions. *AhMYB7* expression was significantly upregulated in BARD479 and PG1102 varieties only after water deficit conditions. Whereas, a significant reduction in *AhMYB7* expression was recorded in the roots of BARI2011, BARI2000, and GOLDEN varieties (Figure 6C). Transparent Testa Glabra 1 (TTG1), which encodes a WD40 domain-containing protein, is required for the accumulation of purple anthocyanin in the leaves. Its involvement has also been reported in the regulation of the flavonoid pathway (Zhang et al., 2003; Gonzalez et al., 2008). This study results indicated that the expression *AhTTG1* is highly stable and tightly regulated in peanut leaves as no significant differences were observed in its expression under optimal growth conditions in the leaves of almost all of the varieties (Figure 5D). However, the water deficit conditions significantly reduced its expression in all the leaves of all the varieties. The lowest reduction in gene expression was recorded in the leaves of BARI2000 variety in response to water deficit conditions, whereas the highest reduction in *AhTTG1* expression was recorded in the leaves of PG1102 and PG1265 varieties (Figure 5D). *AhTTG1* expression profile in roots followed a relatively similar pattern in the roots, where water deficit conditions significantly reduced its expression in all varieties as compared with normal conditions. In roots, the highest *AhTTG1* relative expression was recorded for BARI2000 variety (Figure 6D).

The highest relative expression for *AhbHLH42* was recorded in the leaves of PG1265 and BARD479 varieties under optimal growth conditions, followed by PG1102 and BARI2011 varieties (Figure 5E). Water deficit induced a significant increase in the expression of *AhbHLH42* in the above-mentioned varieties with the highest relative expression recorded in the leaves of BARD479, followed by PG1265, PG1102, and BARI2011 varieties (Figure 5E). The relative expression of *AhbHLH42* in roots followed a relatively different pattern where water deficit conditions induced a significant increase in its expression in BARD479, BARI2011, PG1265, and PG1102 varieties (Figure 6E). On the other hand, the relative expression of *AhbHLH42* was reduced in BARI2000 and GOLDEN varieties after water deficit conditions (Figure 6E). Under optimal conditions, the highest relative expression of *AhGL3* (Glabra 3) was recorded in the leaves of BARD479 followed by PG1102, PG1265, and BARI2011. BARI2000 and GOLDEN varieties had the lowest expression levels of *AhGL3* under normal conditions (Figure 5F). Water deficit conditions significantly reduced the expression of *AhGL3* in the leaves of all the varieties. The lowest expression of *AhGL3* was recorded in BARI2000 and GOLDEN varieties followed by PG1102, PG1265, BARI2011, and BARD479 varieties (Figure 5F). Water deficit conditions had a similar effect on the expression profile of *AhGL3* in roots. Water deficit conditions significantly reduced *AhGL3* expression in roots of all varieties. Maximum reduction in relative expression was observed in roots of GOLDEN variety followed by BARI2000 and BARI2011 varieties (Figure 6F). Water deficit conditions significantly increased *AhMYB123* expression in the leaves of BARI2000 variety only. However, its expression was reduced in all the other varieties in response to water deficit conditions (Figure 5G). In roots, *AhMYB123* expression was significantly increased in PG1265 and PG1102 varieties. However, BARI2000, GOLDEN, and BARI2011 varieties showed a significant reduction in *AhMYB123* expression following the water deficit conditions (Figure 6G). The COP1 suppressor 2 (CSU2) encodes a nuclear coiled-coil domain-containing protein that interacts and co-localizes with COP1. It negatively regulates E3 ubiquitin ligase activity of COP1 (Xu et al., 2015). Maximum relative expression of *AhCSU2* under normal growth conditions was recorded in the leaves of PG1102, followed by BARI2000, BARI2011, PG1265, and BARD479 varieties. The lowest expression was observed in the leaves of the GOLDEN variety. However, the water deficit conditions significantly reduced the expression of *AhCSU2* in the leaves of all varieties except GOLDEN, where the relative expression was significantly increased in response to the water deficit conditions (Figure 5H). On the other hand, the water deficit conditions caused a significant increase in the expression of *AhCSU2* in the roots of the GOLDEN variety only. The rest of the varieties showed a significant reduction in expression of *AhCUS2* following water deficit conditions with maximum reduction recorded for BARI2011, BARD479, PG1102, and PG1265 varieties (Figure 6H).

### Interdependence and Co-dependence Among Selected Variables

Interdependence and co-dependence among selected variables were determined by using the Pearson *r* test. Correlational analysis between the expression of flavonoid biosynthetic pathway genes and TFs in peanuts under water deficit conditions revealed a significantly strong negative correlation between the expression of *AhMYB111* and *AhMYB7* with the expression of *AhF3H* (*r* = −0.8851) and *AhFLS* (*r* = −0.7345), respectively (Table 2). On the other hand, a moderate level positive correlation has been observed between the expression of *AhMYB7* and *Ah4CL* (*r* = 0.6105), *AhTTG1 and AhCHS* (*r* = 0.5073) and, *AhCSU2 and AhF3H* (*r* = 0.5565). *AhbHLH* and *AhGL3* revealed nil-to-little relation with the expression of flavonoid biosynthetic pathway genes. Predominantly, a fair degree of correlation has been observed between the expression of flavonoid biosynthetic pathway genes and TFs under water deficit conditions which indicates coregulation (Table 2).

**Table 2:** Correlational analysis between the expression of flavonoid biosynthetic pathway genes and transcription factors (TFs) in peanuts under water deficit conditions.

**Transcription factors**		**Flavonoid biosynthetic genes**								
		AhC4H	Ah4CL	AhCHS	AhCHI	AhF3H	AhFLS	AhLAR	AhDFR	AhANS
AhMYB111	*r*	0.1617	0.2588	−0.3826	0.1125	−0.8851	−0.193	−0.019	0.209	0.2578
	*p*-value	0.6157	0.4166	0.2196	0.7279	0.0001	0.5478	0.9531	0.5145	0.4185
AhMYB123	*r*	−0.1228	0.4622	0.08268	−0.0164	−0.09097	−0.03508	0.06566	0.1274	0.4621
	*p*-value	0.7037	0.1303	0.7984	0.9597	0.7786	0.9138	0.8393	0.6931	0.1304
AhMYB7	*r*	0.216	0.6105	−0.2779	0.1071	−0.6606	−0.7345	−0.4292	0.2435	0.44
	*p*-value	0.5002	0.035	0.3819	0.7403	0.0194	0.0065	0.1638	0.4456	0.1523
AhTTG1	*r*	0.2065	0.2907	0.5073	0.3145	0.2346	−0.3637	−0.3264	0.4016	0.1361
	*p*-value	0.5196	0.3594	0.0923	0.3194	0.463	0.2452	0.3005	0.1956	0.6731
AhbHLH	*r*	0.2312	0.2305	0.2059	0.1517	0.03581	−0.0737	0.08961	0.2068	0.02192
	*p-*value	0.4696	0.471	0.5209	0.6379	0.912	0.8199	0.7818	0.5191	0.9461
AhGL3	*r*	0.204	0.215	0.2955	0.1424	0.2089	0.0763	0.1633	0.1961	0.1832
	*p*-value	0.5248	0.5022	0.3511	0.6589	0.5146	0.8137	0.612	0.5412	0.5688
AhCOP1	*r*	0.2912	0.2201	0.4931	0.3469	0.1796	−0.2958	−0.152	0.4186	−0.0278
	*p*-value	0.3585	0.4918	0.1033	0.2693	0.5764	0.3506	0.6373	0.1756	0.9315
AhCSU2	*r*	−0.1084	0.2387	0.3486	−0.1247	0.5565	0.4441	0.3365	−0.0895	0.2339
	*p*-value	0.7373	0.4549	0.2668	0.6994	0.0602	0.148	0.2849	0.782	0.4643

*r, Pearson r that is showing a correlation between two variables*.

*p < 0.05 is considered to be statistically significant*.

Correlation of flavonoid biosynthetic pathway gene expression with RWC under water deficit conditions revealed positive correlation of RWC with the expression of *AhF3H* (*r* = 0.6253), *AhCHS* (*r* = 0.4525) in leaves and with the expression of *AhC4H* (*r* = 0.5467), *AhCHS* (*r* = 0.4887), *AhCHI* (*r* = 0.4364), and *AhF3H* (*r* = 0.4153) in roots (Table 3). The accumulation of phenolics in leaves of peanut under water deficit conditions indicated a fair degree of dependence on the expression of *AhC4H* (*r* = 0.4641), *AhCHI* (*r* = 0.4725), and *AhDFR* (*r* = 0.4338) in leaves, whereas the accumulation of phenolics in roots showed a moderate degree of dependence on the expression of *AhCHS* (*r* = 0.5544) and *AhF3H* (*r* = 0.6185) and a strong degree of dependence on the expression of *AhFLS* (*r* = 0.7589) and *AhLAR* (*r* = 0.9651). In leaves, a negative correlation has been observed between the accumulation of flavonol and the expression of *AhC4H* (*r* = −0.6341), *AhCHI* (*r* = −0.6099), and *AhDFR* (*r* = −0.5898) while a positive correlation was detected between the accumulation flavonol and the expression of *AhANS* (*r* = 0.6337), *AhF3H* (*r* = 0.5129). In roots, the expression of *Ah4CL* (*r* = −0.9326) and *AhANS* (*r* = −0.7767) was negatively correlated, while the expression of *AhFLS* (*r* = 0.6073), *AhLAR* (*r* = 0.5357) and *AhF3H* (*r* = 0.5786), *AhCHI* (*r* = 0.6426) was positively correlated with the accumulation of flavonols. The accumulation of anthocyanin in leaves was found to be negatively correlated with the expression of *AhC4H* (*r* = −0.8221), *AhCHI* (*r* = −0.7759), and *AhDFR* (*r* = −0.7769), while the accumulation of anthocyanin in roots was found to be negatively correlated with the expression of *AhFLS* (*r* = −0.6741), *AhF3H* (*r* = −0.4706), and *AhLAR* (*r* = −0.6993). The correlational analysis of the expression of *AhANS* with the accumulation of phenolics, flavonols, and anthocyanins indicated negative correlation except with the accumulation of flavonols in leaves (*r* = 0.6337) and the accumulation of anthocyanins in roots (*r* = 0.5074).

**Table 3:** Correlation of flavonoid biosynthetic pathway gene expression with the accumulation of phenolics, flavonols, and anthocyanins in peanuts under water deficit conditions.

**Biosynthetic Genes**		**RWC**		**Phenolics**		**Flavonols**		**Anthocyanins**	
		**Leaves**	**Roots**	**Leaves**	**Roots**	**Leaves**	**Roots**	**Leaves**	**Roots**
AhC4H	*r*	0.2037	0.5467	0.4641	−0.0842	−0.6341	−0.1261	−0.8221	0.3828
	*p*-value	0.6986	0.2616	0.3538	0.874	0.1763	0.8118	0.0447	0.4539
Ah4CL	*r*	0.2684	−0.2222	−0.285	−0.3072	0.4964	−0.9326	0.5083	0.3275
	*p*-value	0.6071	0.6722	0.584	0.5536	0.3166	0.0067	0.3032	0.5263
AhCHS	*r*	0.4525	0.4887	0.3028	0.5544	−0.1072	0.2343	−0.165	−0.3892
	*p*-value	0.3676	0.3253	0.5596	0.2536	0.8398	0.655	0.7548	0.4457
AhCHI	*r*	0.2403	0.4364	0.4725	0.1381	−0.6099	0.6426	−0.7759	−0.01007
	*p*-value	0.6465	0.3869	0.344	0.7942	0.1986	0.1688	0.0697	0.9849
AhF3H	*r*	0.6253	0.4153	0.2431	0.6185	0.5129	0.5786	0.4077	−0.4706
	*p*-value	0.1843	0.4129	0.6425	0.1905	0.2981	0.2289	0.4223	0.3462
AhFLS	*r*	0.2116	0.06708	−0.0448	0.7589	0.3905	0.6073	0.03341	−0.6741
	*p*-value	0.6874	0.8995	0.9328	0.0802	0.444	0.201	0.9499	0.142
AhLAR	*r*	0.298	0.3954	0.2871	0.9651	0.2077	0.5357	−0.1485	−0.6993
	*p*-value	0.5663	0.4378	0.5811	0.0018	0.6929	0.2734	0.7788	0.122
AhDFR	*r*	0.2488	0.4776	0.4338	0.3911	−0.5898	0.1098	−0.7769	−0.04606
	*p*-value	0.6345	0.3381	0.3901	0.4433	0.2179	0.8359	0.0691	0.931
AhANS	*r*	0.3108	−0.6341	−0.5849	−0.4633	0.6337	−0.7767	−0.1269	0.5074
	*p*-value	0.5488	0.1764	0.2227	0.3548	0.1767	0.0692	0.8107	0.3042

*RWC, Relative water content*.

*r, Pearson r that is showing a correlation between two variables*.

*p < 0.05 is considered to be statistically significant*.

Correlational analysis between expression of TFs related to the biosynthesis of flavonoids and the accumulation of phenolics, flavonols, and anthocyanins revealed coregulation of the expression TFs under water deficit conditions in peanut. A positive correlation has been observed between RWC and the expression of *AhTTG1* (*r* = 0.6102), *AhGL3* (*r* = 0.8594), *AhCOP1* (*r* = 0.6687), and *AhCSU2* (*r* = 0.8468), while negative correlation has been observed between RWC and the expression of *AhMYB111* (*r* = −0.664) in leaves. The accumulation of flavonols in leaves showed a positive correlation with the expression of *AhGL3* (*r* = 0.5525) and *AhCSU2* (*r* = 0.6536), while the accumulation of flavonols in roots showed a negative correlation with the expression of *AhMYB7* (*r* = −0.9788) and *AhTTG1* (*r* = −0.6315). The accumulation of anthocyanins in leaves showed negative correlation with the expression of *AhMYB111* (*r* = −0.5505) while the accumulation of anthocyanins in roots showed a positive correlation with the expression of *AhMYB7* (*r* = 0.5721) (Table 4).

**Table 4:** Correlational analysis between expression of TFs related to the biosynthesis of flavonoids and the accumulation of phenolics, flavonols, and anthocyanins in peanuts under water deficit conditions.

**Transcription Factors**		**RWC**		**Phenolics**		**Flavonols**		**Anthocyanins**	
		**Leaves**	**Roots**	**Leaves**	**Roots**	**Leaves**	**Roots**	**Leaves**	**Roots**
AhMYB111	***r***	−0.664	−0.1943	−0.2821	0.4464	−0.5686	−0.3886	−0.5505	−0.2013
	***p*****-value**	0.1503	0.7122	0.5881	0.3749	0.2391	0.4464	0.2576	0.7021
AhMYB123	***r***	−0.1719	−0.3691	−0.4555	0.3742	−0.1127	−0.3209	−0.06348	−0.0427
	***p*****-value**	0.7447	0.4715	0.364	0.4649	0.8317	0.5352	0.9049	0.936
AhMYB7	***r***	−0.2597	−0.00379	−0.1765	−0.3993	−0.3131	−0.9788	−0.2302	0.5721
	***p*****-value**	0.6192	0.9943	0.7381	0.4329	0.5457	0.0007	0.6608	0.2354
AhTTG1	***r***	0.6102	−0.1282	0.05815	0.2031	0.2044	−0.6315	0.1562	0.2555
	***p*****-value**	0.1983	0.8087	0.9129	0.6996	0.6977	0.1786	0.7676	0.6251
AhbHLH	***r***	0.2723	0.3606	−0.1832	0.4962	0.03258	−0.383	−0.05365	0.09103
	***p*****-value**	0.6016	0.4826	0.7282	0.3168	0.9511	0.4536	0.9196	0.8638
AhGL3	***r***	0.8594	0.07502	−0.2547	0.4262	0.5525	−0.278	−0.00864	0.2119
	***p*****-value**	0.0283	0.8877	0.6262	0.3995	0.2555	0.5937	0.987	0.6869
AhCOP1	***r***	0.6687	0.5747	0.07857	0.2208	0.1704	−0.317	0.02782	−0.4735
	***p*****-value**	0.1465	0.2329	0.8824	0.6741	0.7469	0.5404	0.9583	0.3428
AhCSU2	***r***	0.8468	−0.02953	−0.0556	0.7113	0.6536	−0.1199	0.3178	−0.53
	***p*****-value**	0.0334	0.9557	0.9167	0.113	0.1592	0.8209	0.5394	0.2795

*RWC, Relative water content*.

*r, Pearson r that is showing a correlation between two variables*.

*p < 0.05 is considered to be statistically significant*.

## Discussion

Flavonoids are widely distributed secondary metabolites of plants. Flavonoid biosynthesis includes early pathway enzymes, CHS and CHI, and the late pathway enzymes, F3H, FLS, DFR, C4H, 4CL3, and LAR (Winkel-Shirley, 2001). Flavonoids are chemical scaffolds that have properties like typical ROS scavengers, which highlights their significance under stress conditions. Flavonoid production under stress has been reported in different crops (Christie et al., 1994; Ithal and Reddy, 2004; Yuan et al., 2012; Liu et al., 2013; Ma et al., 2014). Similarly, various stress conditions have also been shown to alter the expression of flavonoid biosynthetic genes.

Drought is a hostile environmental condition that has the potential to reduce the production of arid and semiarid land crops, such as peanuts. Several studies have indicated the role of drought on the expression and accumulation pattern of flavonoids (Ma et al., 2014). This study elucidated expression characterization of flavonoid biosynthetic pathway genes and TFs associated with flavonoid production under water deficit conditions in six peanut varieties showing differential response against water deficit conditions. As drought is technically a long-term phenomenon, investigations into the expression patterns of genes involved in delayed responses to water deficit conditions indicated the induction of genes such as *FLS, LAR, DFR, ANS, 4CL3*, and *C4H*. Although, in some of the varieties the expression of late pathway genes was downregulated, the overall expression of the biosynthetic genes was elevated especially in those varieties which were found to be drought tolerant. These results are in accordance with the previous studies conducted in tea plants (Zheng et al., 2016).

Water shortage for the short and/or long term drastically affects the root system, as it is the first point of contact. That is why, the expression pattern of both early and late pathway genes in roots of peanut showed a marked variation from expression in leaves. Moreover, correlational analysis and accumulation pattern of phenols, flavonols, and anthocyanins confirmed the differential response in leaves and roots to water deficit conditions. Studies have shown that the expression of flavonoid biosynthetic genes is upregulated in the roots of Arabidopsis plants as compared with the leaves (Nguyen et al., 2016). Moreover, it has also been reported that drought stress induces the expression of flavonoids biosynthesis genes in the roots of *Scutellaria baicalensis Georigi* (Yuan et al., 2012).

The expression of *AhMYB111* showed a more pronounced increase in the leaves as compared with roots following water deficit conditions. Variable transcriptional responses of different TFs to abiotic stress have been reported earlier. Nakabayashi et al. (2014) reported that overexpression of MYB enhances oxidative and drought tolerance in Arabidopsis by over-accumulation of antioxidant flavonoids (Nakabayashi et al., 2014). Furthermore, the expression of *AhCOP1* was found to be downregulated under water deficit conditions in all the varieties. Constitutive Photomorphogenesis 1 (COP1), a photomorphogenesis repressor with E3 ubiquitin ligase activity, is a key regulator of plant growth in response to light (Kim et al., 2017). The drought-induced general reduction in *AhCOP1* expression is also reported by Liu et al. (2018), who reported generally low and decreasing expression patterns of photoreceptor genes including *AhCOP1* as compared with TFs including *MYB111* involved in flavonol biosynthesis under different light regimes. The Arabidopsis, *AhMYB7*, encodes a transcriptional repressor of flavonol biosynthesis (Stracke et al., 2010), and its maximum relative expression was recorded in BARI2000 and GOLDEN varieties following the water deficit conditions, which explains the lowest levels of flavonols accumulated in the leaves and roots of these two varieties. In plants, the bHLH42 also known as the Transparent Testa 8 (TT8) together with a member of the MYB TF family positively regulates the biosynthesis of anthocyanin (Wang et al., 2019). bHLH42 and MYB123 physically interact to activate the dihydroflavonol reductase (DFR) promoter (Baudry et al., 2004). The TT8 locus is also involved in the regulation of flavonoid biosynthesis in Arabidopsis (Nesi et al., 2000). The combined role of bHLH42 and MYB123 in plants has been well-investigated and is more well-known in plant responses to thermal stress (Wos, 2016), salt stress (Jiang et al., 2009), and flavonoid biosynthesis (Pucker et al., 2020). Similarly, another bHLH TF GL3 that primarily interacts with GL1 for trichrome development has also been shown to be required for anthocyanin accumulation and is induced by nitrogen depletion (Feyissa et al., 2009). In this study, water deficit conditions significantly reduced GL3 expression in the leaves and roots of the peanut varieties. MYB123, also known as the TT2 in Arabidopsis, encodes an R2R3 MYB domain-containing TF which is a key determinant in the proanthocyanidin accumulation and has been shown to upregulate flavonoid biosynthesis (Ravaglia et al., 2013). In this study, the water deficit conditions induced a significant increase in its expression in the leaves of the BARI2000 variety only. But its expression was significantly reduced in the roots of the same variety. This may be due to differential tissue-specific transcriptional regulation of MYB123.

Moreover, the accumulation of flavonoids was upregulated in leaves and roots after drought treatment and the GOLDEN variety depicted higher accumulation as compared with its respective control and other varieties. It is speculated that flavonoids may achieve their antioxidant function by blocking the production of ROS and scavenging cellular ROS (Agati and Tattini, 2010). The protective function of anthocyanin under different stress conditions is based on the quantity of anthocyanin and the expression of associated genes (Nakabayashi et al., 2014; Singh et al., 2015). It is, however, obvious that the accumulation of anthocyanin during stress response would also be dependent upon the varieties. In the current study, water deficit conditions increased the accumulation of anthocyanin in peanut varieties which was consistent with the findings of von Wettberg et al. (2010) and Liu et al. (2013). Ma et al. (2014) also reported a significant increase in the accumulation of anthocyanin, phenolics content, and flavonols following water deficit conditions. Phenolic compounds in plants are affected by various environmental stresses (Lee et al., 2012; Zhang et al., 2015). In this study, the phenolic content was increased under drought stress and this increase was more in PG1102 and GOLDEN varieties. Because of the protective effects of phenolic against photoradiation (Takahashi and Badger, 2011) and their function as a kind of non-enzymatic antioxidant during drought stress (Quan et al., 2019), the higher accumulation of phenolic compounds in plants following drought stress, may be a mechanism for the protection of the photosynthetic machinery of peanut leaves under drought stress.

Leaf RWC is another important physiological feature that is widely used to define the sensitivity of plant to tissue and cell dehydration. Several studies have reported that the minimum reduction of RWC under water deficit stress indicates stress resistance (Hussain et al., 2019; Pour-Aboughadareh et al., 2019). In our study, BARI2011 has maximum RWC under water deficit conditions as compared with other varieties. These results are consistent with the findings of Wang et al. (2008), who reported that water deficit can also lead to a decrease in RWC in wheat (Hu et al., 2010).

Correlational analysis revealed interdependence and co-dependence among selected variables under water deficit conditions. *AhMYB111* and *AhMYB7* act as an inhibitor for *AhF3H* and *AhFLS*, respectively. Whereas, *AhMYB7, AhTTG1*, and *AhCSU2* are positive regulators for the expression of *Ah4CL, AhCHS, and AhF3H*, respectively. *AhbHLH* and *AhGL3* revealed nil-to-little relation with the expression of flavonoid biosynthetic pathway genes. Predominantly, a fair degree of correlation has been observed between the expression of flavonoid biosynthetic pathway genes and TFs under water deficit conditions which indicated coregulation. Their roles still need to be characterized through functional genomics.

Correlational analysis between expression of TFs related to the biosynthesis of flavonoids and the accumulation of phenolics, flavonols, and anthocyanins indicated coregulation of flavonoid synthesis by TFs under water deficit conditions in peanut. *AhTTG1, AhCOP1, AhGL3, AhCOP, AhMYB111*, and *AhCSU2* might be the regulators to generate drought response as their expression showed a moderate degree correlation with RWC. *AhGL3* and *AhCSU2* indicated their role as positive regulators and *AhMYB7* and *AhTTG1* indicated their role as negative regulators in the accumulation of flavonols. The role of *AhMYB111* as a negative regulator has also been observed in the accumulation of anthocyanins. *AhMYB7* indicated its role as a positive regulator in the accumulation of anthocyanins.

## Conclusion

Peanuts, other than containing beneficial fatty acids and micronutrients, harbor various bioactive compounds. Among them, flavonoids hold a prominent position as they are potential antioxidants in plants and protect them from oxidative stress. Globally, breeders are trying to develop peanut varieties as functional food with enhanced flavonoid contents. Peanuts were reported to possess various forms of flavonoids including C-glycoside flavone, flavonol, dihydroflavonol, flavonone and 5,7-dimethoxyisoflavone, and dihydroquercetin. Understanding of the flavonoid biosynthetic pathway in peanuts can open many horizons. In this study, expression characterization flavonoid biosynthetic pathway genes and TFs along with the accumulation pattern of selected flavonoids have been conducted to unravel the significance of flavonoid biosynthetic pathway in peanuts under water deficit conditions. Six varieties of peanuts (BARD479, BARI2011, BARI2000, GOLDEN, PG1102, and PG1265) were selected based on their yield potential and their performance against drought stress. Among them, BARI2011 is the best drought-tolerant variety. According to the given study, higher water retention has been observed in BARI2011. Moreover, a higher accumulation of phenols, anthocyanins, and flavonols has been observed in both leaves and roots while most of the flavonoid-related genes were downregulated or at a normal level which indicated the maintenance of normal homeostasis. Generally, expression analysis, correlational analysis, and accumulation pattern of phenols, flavonols, and anthocyanins only confirmed the differential response in leaves and roots to water deficit conditions. Furthermore, the study confirmed that the biosynthesis of flavonoid in peanut depends upon the genotype of the variety in spatio-temporal manner in water deficit conditions. The study would provide an insight into the role of flavonoid biosynthetic pathway in drought response in peanut and would aid to develop drought-tolerant varieties of peanut. Moreover, the study provides reference data for the flavonoid biosynthetic pathway engineering for higher production of flavonoids for industrial use.

## Data Availability Statement

The original contributions presented in the study are included in the article/Supplementary Materials, further inquiries can be directed to the corresponding author/s.

## Author Contributions

The project was conceived by RA and GK. RA, DC-R, GK, MK, TS, and AH were involved in the planning of the experiments. GK and MK conducted the experiments and collected the data. TS and MK performed data analysis. GK, MK, FM, TS, AH, AG, and RA drafted the manuscript. DC-R, RA, and AG revised the final manuscript. All authors contributed to the article and approved the submitted version.

## Conflict of Interest

The authors declare that the research was conducted in the absence of any commercial or financial relationships that could be construed as a potential conflict of interest.
